# Parents’ and teachers’ views of the promotion of healthy eating in Australian primary schools

**DOI:** 10.1186/s12889-021-11813-6

**Published:** 2021-10-05

**Authors:** Gozde Aydin, Claire Margerison, Anthony Worsley, Alison Booth

**Affiliations:** 1 Institute for Physical Activity and Nutrition (IPAN), School of Exercise and Nutrition Sciences, Deakin University, Geelong, Australia; 2 School of Exercise and Nutrition Sciences, Deakin University, Melbourne, Australia

**Keywords:** Primary school, Health promotion, Parents, Teachers, Qualitative methods

## Abstract

**Background:**

Primary schools have long been identified as appropriate settings for improving the healthy eating behaviours of children and helping them develop food skills. This qualitative study explored the views of Australian primary school parents and teachers about schools’ strengths and weaknesses in promoting healthy eating and equipping children with food skills.

**Methods:**

Nineteen parents and 17 teachers from Victoria participated in semi-structured interviews. Audio recordings were transcribed and underwent thematic analysis using Nvivo.

**Results:**

This study demonstrated that parents and teachers believed that several facilitators helped promote children’s healthy eating. These included food and nutrition education (FNE) programs, the community-based nature of schools, and teacher role modelling and the authority schools possess over children. Time scarcity, lack of teacher expertise, lack of leadership and funding were reported as barriers. School food environments such as canteens, lunch orders, fundraising events and school fairs were identified as both weaknesses and strengths by parents and teachers, which indicated inconsistent implementation of school nutrition policies across schools.

**Conclusions:**

Australian primary schools demonstrate some useful efforts to promote healthy eating among children. However, there are numerous facilitators and barriers which impact on the promotion of healthy eating. These factors need to be addressed in order to develop healthy eating habits further among elementary students. These results provide directions for policymakers and school managers, as they point to the areas that need to be improved to assist the design of schools that better promote healthy eating among children.

**Supplementary Information:**

The online version contains supplementary material available at 10.1186/s12889-021-11813-6.

## Introduction

Healthy practices established at early ages, such as following a well-balanced diet with adequate amounts of fruit and vegetables, have the potential to last a lifetime [1, 2]. These eating habits reduce the prevalence of diet-related diseases such as type 2 diabetes and cardiovascular disease later in life [3, 4]. One key setting to support the well-being of children can be primary schools. Therefore, the compulsory primary school years may provide a useful starting point for the promotion of healthy eating among children [5, 6]. Primary schools provide opportunities to reach a large segment of children, their families, and the wider community regardless of their socioeconomic status and, therefore, help ensure equitable health outcomes [7]. In addition, children consume approximately one-third of their daily meals at school [8–10]. Schools can impact children’s diet through curriculum content, changes to the school physical and policy environment and interaction with parents and the local community [11].

Australian government regulatory departments and health authorities also recognise primary schools as key environments for addressing public health issues among children [12]. To date, both Federal and States Governments have made an effort to promote healthy eating through primary schools. All states have a school food policy. However, compliance is usually voluntary, and procedures for monitoring and evaluation are absent [13]. There have also been some small-scale school healthy eating interventions conducted in partnership with local governments such as the Tooty Fruity Vegie project (ten primary schools, 1 year), Fresh Kids (four primary schools, over 4 years), and Be Active Eat Well (six primary schools, over 3 years) [14–16]. In addition, some aspects of food and nutrition education (FNE) are addressed in the Australian curriculum [17] as well as in the curriculum of the State of Victoria [18]. However, the extent to which the curriculum is translated into classroom teaching is unclear.

A significant barrier for public policymakers implementing interventions and programs in schools is the likely acceptance of these by stakeholders [19]. This emphasises the importance of obtaining stakeholders’ views on school nutrition interventions to better develop and execute future interventions [19, 20]. The views of parents and teachers are essential for both the successful implementation of school FNE programs and policies that involve the cooperation of these two major stakeholder groups [20–22].

To date, there has been limited research on Australian teachers and parents’ opinions about school food practices and school food environments and how these practices and environments can effectively influence the dietary habits of children. Parents’ and teachers’ views have been investigated in a limited number of studies, for example, on school health interventions (Health4kids, DELISH) [23, 24], school canteens [25, 26], kitchen and garden programs [27, 28] and on FNE in Victorian curriculum [29]. However, this descriptive study provides an integrated exploration of all aspects of school food issues, including FNE, food environments and food policy in primary schools. We, therefore, explored the views of teachers and parents on Australian primary schools’ strengths and weaknesses in promoting healthy eating and developing food skills.

## Methods

The authors conducted two separate qualitative studies, one with parents and one with teachers concerning their views of food and nutrition in primary schools. This paper reports on parents’ and teachers’ responses to a single question: *What strengths and weaknesses do you think primary schools possess in promoting healthy eating?* The methodology for both studies is outlined below.

Social constructivism formed the foundation of this study [30]. A qualitative descriptive approach [31] was adopted. In qualitative descriptive studies, descriptions from research participants’ viewpoints help present the facts of the phenomenon in everyday language [32]. Specifically, the questions asked of participants in both studies were broad to lessen the influence of the researcher [33].

### Participants and recruitment

Parents and teachers from all types of primary schools (government, independent, and Catholic) were purposefully [34] recruited from Melbourne suburbs and regional areas of Victoria. The inclusion criterion for parents was having a child attending a primary school in Victoria. For teachers, it was having taught any aspect of nutrition in a primary school in Victoria. Advertisements for the study were circulated on Facebook groups for parents and teachers, and notices were posted on neighbourhood notice boards at libraries and supermarkets. A snowball sampling strategy [35], where participants assisted researchers to identify other potential participants [school type and geolocation], also facilitated the recruitment process. Thirty-seven parents and 26 teachers responded to the advertisement via emails. Parents and teachers were asked the name and suburb of the school to ensure that no more than one parent/teacher was included from any particular school to achieve maximum variation of school type. When selecting participants, the type of school was recorded to ensure that parents and teachers represented government, Catholic and independent schools which included religious schools such as Christian, Islamic and Jewish schools. After scheduling the first ten interviews with parents and teachers from government schools, priority was given to the school types that were less represented in the sample when planning further interviews.

All the selected parents and teachers were provided with a plain language statement and consent form via email before each interview. Parents and teachers who were not selected were informed by email. The selected participants gave their written consent to participate and permission to audio-record the interviews. They were all offered a $20 shopping voucher as compensation for their time. There were no prior relationships between the researchers and the participants. The recruitment ceased when the ‘conceptual depth’ (data saturation) point was reached, which is in the words of Nelson: ‘ a *sufficient depth* of understanding that can allow the researcher to theorise’ [36].

### Interview *procedure*

Semi-structured qualitative interviews were conducted to obtain a detailed understanding of Australian parents’ and teachers’ views on the Victorian primary schools’ strength and weaknesses in promoting healthy eating. Individual interviews were conducted by the lead researcher (GA) either face-to-face (*n* = 15) or via phone (*n* = 21). Face-to-face interviews were conducted in public locations such as libraries or participants’ residences. The duration of the parental interviews ranged between 22 to 54 min, with an average of 37 min. For the teachers, interviews ranged between 28 to 62 min, with an average of 47 min. It should be noted that these reported interview durations included participants’ responses to four questions, only one of which is reported in the present paper (see above).

The interview questions were original and based on a review of the literature. Face validity assessment of interview questions was made through two pre-test interviews for each group of participants. The pre-tested data was combined with information obtained from the later interviews as only minor phrasing changes were made in the questions after the pre-testing. Parents and teachers were asked four broad, open-ended questions using an interview guide. This paper presents the analysis of responses from one of these broad questions, as noted above.: General prompts from the interviewer included “Could you tell me more about that?”, “Is there anything else?”, “Why?”, “What do you mean by …” ,“How …? ” / “Where …? / “What for …? to let participants explain more.

### Data analysis

The interviews were digitally recorded and transcribed by an external contractor (Rev.com) verbatim. All transcripts were checked for accuracy by the lead author. Parents and teachers were given the chance to review the transcripts of their interviews. Only one teacher opted to review her interview but did not request any changes.

Interview transcripts were manually coded using the qualitative data analysis software package NVivo (SR International Pty Ltd. Version 12, 2015). The lead researcher read the transcripts several times to familiarise herself with the data. King’s template analysis method [37] was used for data analysis which commenced immediately after transcription of the first two interviews. An initial template with a priori codes was created based on the research question. This was used to expedite the initial stage of interview coding [37]. New codes were then inserted into the initial template during the remaining data coding process. The codes identified by GA and research context were discussed with other researchers of the study for the accuracy of interpretation during weekly meetings [38, 39]. The final template comprising the themes was reviewed by all authors and is described in the results section below.

To enhance the trustworthiness of the findings, themes generated by the manual coding using Nvivo were compared with those generated by Leximancer [40, 41]. Leximancer (www.leximancer.com) is a machine learning-based qualitative data analysis software that enables fast visual generation of themes and related concepts automatically derived from qualitative data [42, 43]*.*

## Results

Nineteen parents of children attending a primary school in Victoria from the suburbs of Melbourne and regional areas of Victoria were interviewed. Despite their diverse ethnic backgrounds, all parents had lived in Australia for at least 6 years. They all had post-secondary education and reported working at least part-time. They all had at least one child (aged 5–12 years old) enrolled in a primary school in Victoria. Seventeen teachers who worked or have been working in a primary school in Victoria with a range of teaching experience were interviewed. Teachers were recruited from the suburbs of Melbourne and regional areas of Victoria (Table 1). All interviews were held from January 2020 to June 2020. Identified themes were given in Table 2. The same themes were identified in both parents’ and teachers’ interviews.

**Table 1:** Demographic characteristics of participants

	*Parents*	*Teachers*
	*(n = 19)*	*(n = 17)*
**Gender**		
Female	18	12
Male	1	5
**School type**		
Government	11	13
Independent	5	3
Catholic	3	1
**Education**		
PhD	2	–
Masters/Graduate Diploma	5	6
Bachelor	8	11
Diploma	4	–
**Geolocation**		
Melbourne (major city)	17	16
Regional areas of Victoria	2	1
**Ethnicity**		
Australian	6	15
Other^a^	13	2
**Teaching experience (in years)**		
0–5	NA	6
5 to 10 years		3
10 to 15 years		3
15–20		2
> 20 years		3

^a^‘Other’ denotes parents from Chilean, Turkish, Israeli, Japanese, Indonesian, New Zealander, Singaporean, Italian, Vietnamese, Polish, Persian, Swedish, Taiwanese backgrounds. Teachers from non-Australian backgrounds were from Indian and Turkish backgrounds

**Table 2:** Themes identified in the study

**Perceived strengths of primary schools in the promotion of healthy eating**	
Theme 1. Food and nutrition education programs	
Theme 2. Being community based, reaching parents	
Theme 3. Teachers as role models and the authority of schools	
Theme 4. Healthy school food environments	
a. Fruit breaks and providing free fruit	
b. Rules over food breaks	
c. Healthy canteens and lunch orders	
**Perceived weaknesses of primary schools in the promotion of healthy eating**	
Theme 1. Limited time	
Theme 2. Lack of knowledge, expertise and motivation	
Theme 3. Unhealthy school food environments	
Theme 4. Cost of food and nutrition education and associated lack of funding	

### Perceived strengths of primary schools in the promotion of healthy eating

#### Food and nutrition education programs

Teachers (*n* = 9) and parents (*n =* 5) discussed the impact of school FNE programs and related curricula on children’s eating habits and skills. They believed these programs, especially ones with a practical component, have a positive impact on children.

*‘I think probably the Stephanie Alexander kitchen* [An independent kitchen and garden program offered in some Australian primary schools] *is a huge thing. That’s really one of the reasons I enrolled the children at that school because of this program. So, they grow the fruit or food, vegetables, and then they pick it and then they cook it and then they eat. So, it’s right from the start really.’ Parent 18.*

*‘I think that for my school specifically, being able to give them that cooking program and the gardening that is something that is a massive strength for them, because they learn all these different things and it’s so good for them because they get the chance to do things they might not be able to do at home.’ Teacher 3.*

Primary school children’s young age and the significant amount of time spent at school were also cited as advantages that contribute to the effectiveness of FNE delivered at primary schools. Some parents (*n* = 2) and teachers (*n =* 2) emphasized the importance of teaching FNE from a young age, which can lead to behaviour change amongst primary school children.

*‘They are at their optimal learning. So, for kids to learn something, that can be weaved across six years of learning in primary school. So, we have time, and we have the minds that are ready to learn those things. If you tell this to a 50-year-old, I’m sure they’re going to struggle to try and change their diet and habits. Whereas, a kid, they’re very willing to take on that advice from someone they trust.’ Teacher 13.*

*‘I think getting in while they’re young, it’s something they can remember. Teach them early so they don’t forget, or they don’t think of as an additional tool. It becomes part of their routine.’ Parent 19.*

*‘I guess they have the kids for six hours a day, five days a week. They have a captive audience.’ Parent 12.*

#### Being community-based, reaching parents

Some teachers (*n* = 6) and parents (*n =* 4) believed that primary schools were in a position to reach parents too and provide them with information and support in feeding their children healthily. Both groups claimed that as parents play a key role in their children’s eating habits, it is crucial to target parents as well as children in any initiative to improve effectiveness.

Some participants suggested that schools might send informative brochures to parents or provide opportunities to join practical FNE activities running at schools, such as cooking or gardening classes or cultural food days.

*‘When my little boy starts prep* [first year of primary/elementary school] *this year, the parents are provided with a brochure about healthy eating and how to prepare lunchbox, a healthy lunch box for the kids, and they give us a lot of ideas for the lunchboxes, which is good. It’s quite scientific and quite a lot of ideas’ Parent 18.*

*‘I think because primary schools are very community based, I feel like that is one of their strengths. Just that there’s so much buy-in from parents, and parents will come and help out. I guess, communication with parents too, in newsletters etc. Parents take on that advice that’s sent out. And the fact that the parents are more engaged in their students’, education at the time. Not to say that they’re not in high school (secondary school) but just that it’s more of a community feel. So, what is being taught in the classroom is then being replicated at home, or being used at home, I guess.’ Teacher 16.*

#### Teachers as role models and the authority of schools

Parents (*n* = 4) and teachers (*n =* 4) mentioned the importance of role modelling, and they recognised the importance of teacher role modelling of healthy eating as a positive influence on children’s eating habits. They believed that teachers have the potential to model healthy eating during recess and lunchtime.

*‘I think modelling it to students can be helpful. So, when they get their snacks out, I’ll get my banana out and start eating it, so they can see, “Oh, he’s eating something healthy,” not overtly saying to them, “Here’s your healthy option,” they’ll just pick up that’ Teacher 1.*

*‘So, you try and model what is the best way of how I would eat, and I’m hoping you all do that too. If you don’t have any fruit in your lunchbox today, it would be wonderful if you have some tomorrow. So, then the child almost nags their mom. The next day there’ll be a piece of fruit in the lunch box.’ Teacher 2.*

The authority that schools possess over children was also discussed, specifically by parents. For example, two parents commented:

*‘A parent can nag the children for 10 years, and they won’t ever listen to anything we’re saying. The school says it once, and they believe it straight away’ Parent 19.*

*‘I guess they’re in a position of authority, just as much as parents are, and I think maybe more so, to be able to impart that type of information on students.’ Parent 12.*

#### Healthy school food environments

##### Fruit breaks and providing free fruit

Participants mentioned fruit breaks that are also named as ‘crunchy fruit time’ or ‘brain food’ when children are given 10 mins to eat either a fresh vegetable or a fruit brought from home or available at the school. Some participants, particularly teachers (*n* = 6), felt that having fruit breaks and providing free fruit to children were among the primary schools’ strengths in promoting healthy eating. They recognised it as an effective strategy to increase the fruit or vegetable consumption of children. Some reported that fruit baskets were usually available for children at fruit breaks (morning break before the lunchtime). These fruit baskets were often donated by charities or nearby supermarkets. One teacher reported that he voluntarily paid for a bag of fruit few times when no fruit was donated, as he noticed that some of his students did not have any fruit in their lunch boxes. Some teachers also added that they encourage children to have fruit at lunchtime if they wanted to.

*‘Last year we organized a partnership with Woolworth’s* [A major supermarket chain], *and they provided a box of bananas and apples each week so then the kids that didn’t have brain food could then have some. I’ve started bringing in like a bag of apples each week, then for mostly one particular student in my class that doesn’t have a very healthy, balanced diet in his lunch box, and so I’ll just give him an apple at recess. So, I think that would be quite common amongst teachers to bring in some food of their own to share for those kids not bringing healthier.’ Teacher 1.*

*‘The idea of this crunchy fruit time where there’s a special time where they bond, they have a little bit of a laugh while eating their crunchy fruit, because it has to be some kind of fruits or vegetables, I think that’s a very good example of how it can be done, how it can be promoted.’ Parent 3.*

##### Rules over food breaks

Some participants (*n* = 6) believed that implementation of policies is one of the facilitators of the promotion of healthy eating. The interviews revealed that many schools have the ‘nude food policy’ for lunch boxes (i.e. packed products are forbidden), although the implementation frequency differed among primary schools. Some schools asked students to have a package-free lunch box once a week, whereas others aimed to apply the policy more often. Parents, in particular, regarded implementation of this policy as a significant effort in promoting healthy eating as well as teaching children to live more environmentally friendly.

*‘ With strengths, it’s nude food. The kids also say that that’s better for the environment which gets them thinking about buying things from the supermarket …*. I *think they’re trying to make it all the time, but they have it two days a week, where it’s supposed to be only nude food. It makes you more conscious of your choices, you have to be a bit more creative.’ Parent 8.*

Not allowing kids to have any fizzy drinks or sugar-sweetened beverages in their lunch boxes was also listed as one of the positive practices of the schools.

*‘I think if the kids were probably bringing cans of soft drink or something like that to school, they would probably pick up on things like that. They’re very pro water’ Parent 16.*

##### Healthy canteens and lunch orders

In Australia, not all primary schools have a school canteen or lunch order option, and some have it for only a few days a week. Most participants believed that having a canteen with a healthy menu at school affects their children’s food choices positively.

*‘For his school compared to others, I think they’re doing a great job. Generally having a canteen at school, I think is a really good thing. So, I was surprised when they introduced that once a week. And from time to time, they do have another day just to provide sushi or something. They are trying to be as healthy as possible.’ Parent 10.*

*‘I know that their school canteen is amazing. It was once featured in The Age* [a daily newspaper in Melbourne] *because it’s such a healthy canteen. So, they have a really lovely canteen there; tuck shop. You can buy lunch there or snacks. It’s all homemade. They make salads and homemade pizza, but there’s nothing deep fried or anything like that.’ Parent 15.*

### Perceived weaknesses of primary schools in the promotion of healthy eating

#### Limited time

A lack of time and other competing priorities of teachers were regarded as a barrier to promoting healthy eating among children, particularly by teachers (*n* = 10). Teachers claimed that they could not find available time to focus on formal or informal FNE teaching opportunities, and the overcrowded curriculum was often mentioned. Unlike teachers, parents did not mention time as a limitation, except for three parents.

*‘I would think that sometimes we had a very overcrowded curriculum and maybe it gets watered down and health and physical ed part of the curriculum is not done as thoroughly as it could be done.’ Teacher 8.*

*‘They’ve got STEM* [science, technology, engineering and mathematics], *library, arts; there are so many other things they have to like fit in’ Parent 13.*

#### Lack of knowledge, expertise and motivation

Almost half of the parents and teachers listed lack of knowledge and/or motivation to teach FNE as a barrier in primary schools. Two teachers also mentioned that the lack of teaching resources negatively impacts teachers’ confidence and knowledge.

*‘Lack of knowledge within teachers, maybe them not feeling confident. I think that’s all down to resources. I know that whenever I’ve done a cooking activity at school, it’s always I’ll go and buy it all out of my money.’ Teacher 11.*

*‘I think the limitation is just the lack of knowledge. I don’t know if they know about a healthy lifestyle, healthy eating.’ Parent 3.*

Moreover, some parents and teachers believed primary schools need specialist teachers to teach FNE.

*‘I don’t think the teachers though are trained as a nutritionist, or a dietitian. They’d have to get a specialist teacher as well in there. I think even the basics, or whether they get somebody in live meetings.’ Teacher 13.*

In addition, many teachers and several parents thought that they need supportive school authorities or a dedicated teacher who takes the leadership role to ensure relevant policies and rules were implemented and actions taken were consistent.

*‘You need your leadership to back you. The leadership is a weakness ultimately because they can turn around and go, “That’s not worth the effort,” and they won’t support it as well as what they could be. You might just be on your own.’ Teacher 14.*

*‘Unless the principal or the teacher is very on board, it’d be very hard to push for a consistent routine.’ Parent 19.*

#### Unhealthy school food environments

Many parents (*n* = 10) and some teachers (*n* = 4) believed unhealthy school environments were among the barriers to promoting healthy eating at schools. They felt that these environments undermine health messages given in the classes. They mentioned the impact of the availability and accessibility of foods in the school environment on children’s eating behaviours. Some parents (*n =* 4) and teachers (*n =* 4) criticised school canteens for promoting unhealthy eating among children. Some parents even expressed their gratitude for not having a school canteen. They believed canteens do not resonate with the healthy eating messages given at school.

*‘They ask us to bring just water to drink. That is important, very good, but they sell some of these milk, with flavour, that also has sugar. You can’t bring the milk in your lunchbox, but you can buy it from the canteen. And you can buy the other things that are in plastic. They even encourage only one day to not bring the things in plastic.’ Parent 3.*

*‘ It’s usually hot dogs and highly processed things and lots of ice creams. So, I would say that would be a weakness, that’s a frustration. We do all this focus in class and in the kitchen and garden, and then it’s sort of like, “Here’s your canteen list, take your pick of all the foods we’re saying “sometimes food”*. ’ *Teacher 1.*

It is important to note that a few parents (*n* = 4) believed that canteen food could be offered infrequently as a treat (e.g. once a week). They were not highly critical of the menus and did not believe the inclusion of more healthy options was a priority.

*‘To me the lunch order is more of a treat, or a Friday type thing. So my kids have hotdogs or chicken nuggets. My kids are not going to have a salad for lunch. they’ve been there a few years they know what the canteen has, all of a sudden if canteens bring in something, they might be a bit like, “No, I just want my hotdog.”’ Parent 13.*

Some parents (*n* = 4) believed birthdays in primary schools cause their children to be exposed to high amounts of sugar. However, two teachers reported they sent the birthday lolly bag or cake brought by the birthday child home to be eaten after school hours thus, they did not list that as a barrier to healthy eating.

*‘Letting kids bring lollies for their birthdays. There’s so many birthdays in classes. And even if they have their birthday on the school holidays, then they have to bring cupcakes on the Friday before the school holiday. You can’t escape any of this. Everyone can bring their own children as much lollies as they want, but do we really need them handed out at school? It’s hard for the kid too, even if they don’t want it. There’s peer pressure, so they will eat it.’ Parent 1.*

School fundraising events were also widely criticised for undermining health promotion at schools. In particular, parents expressed their displeasure with chocolate drives during fundraising efforts.

*‘They’ re trying to raise funds by selling brownies. So, I think in that regard, that does have a negative impact. It would be good to be able to replace some of those fundraising events with healthier options. For example, because in a fundraising event, there were rum balls* [a sweet treat]*. So, instead of rum balls like maybe it could be like protein bars or protein slices.’ Parent 2.*

‘*We did one a couple of years ago with Krispy Kreme* [a doughnut company]*, and the parents didn’t like it, they didn’t want their kids having that. So, we actually didn’t raise a lot of money, because the parents didn’t want them eating that food. So, that was actually a really good indicator for us that the parents would prefer something healthier.’ Teacher 3.*

Parents also directed similar criticism to schools’ events for special days.

*‘I think some of the weaknesses are when they have a school activity, like if they do a sports day or carnival, it’s always a barbecue, sausages and bread, which I don’t like. I think there should be other alternatives, and I know it’s easy to do, but I think, having an activity where you could have something a bit more nutritious, and the kids could participate, would be great.’ Parent 8.*

#### Cost of FNE and associated lack of funding

The cost of providing high quality or holistic FNE was also regarded as a barrier to promoting healthy eating, particularly by government school teachers (*n* = 5). They claimed that they could not run cooking and gardening activities or take children on farm or supermarket visits because of financial restraints. One teacher also mentioned that they could not invite an expert speaker because their school could not afford the cost.

‘*Government primary schools are always trying to find ways to bring in more money and the funding to do the things that the teachers would really like to do. There is a very big difference between schools across Victoria and across Australia depending on the category of government or independent schools. But there’s also a difference by postcode or suburb.’ Teacher 4.*

*‘I think it comes down to cost. A lot of independent schools can have a stand-alone program where they can give their students all these different things, but government schools can’t do that.’ Teacher 3.*

Identified themes derived from the NVivo analysis were confirmed by a Leximancer analysis which enhances the credibility of the findings. (Supplementary file).

## Discussion

As far as we are aware, this is the first qualitative study that has specifically examined parents’ and teachers’ views on the strengths and weaknesses of primary schools in promoting healthy eating. In the study, parents and teachers identified a variety of primary schools’ strengths and cited several areas that need improvement or change.

### Schools’ potential role in promoting healthy eating

Parents and teachers identified what they saw as the main contributors to school’s promotion of healthy eating. These included FNE programs, schools’ ability to reach the wider community, particularly parents, and teacher role modelling of healthy eating.

Most parents and teachers acknowledged the delivery of FNE as crucial and beneficial for children, in particular programs with a practical component such as kitchen and garden programs. Previous studies have demonstrated similar levels of support among parents [44] and teachers [29, 45, 46] for increasing the inclusion of FNE in primary schools. In accordance with the findings of the current study, some other Australian studies have reported parents’ and teachers’ satisfaction with school kitchen and garden programs when classes aimed to influence children’s willingness to taste healthy food [27, 28]. There have also been quantitative studies that have demonstrated minor but significant effects of school gardens on children’s eating behaviours, especially on vegetable preference and intake [10, 47]*.* A recent systematic review of 26 studies from various countries identified the key factors for the effectiveness of primary school FNE interventions in achieving stated objectives [48]*.* These factors included parental engagement, targeting specific behaviours; teacher training or recruitment of trained experts; more time allocated to FNE and age-appropriate activities [48]. Hence, primary schools need to consider these factors to improve the efficiency of FNE.

The present interviewees mentioned the community-based nature of schools and believed parental engagement is critical when promoting healthy eating practices among children. Parents and teachers placed a high value on an effective communication from schools similar to those Australian parents interviewed after the implementation of the HealthLit4Kids program in primary schools [24]. Parents in Western Australia also reported that their children’s diets improved more when information was disseminated to them along with the healthy eating initiatives in schools [49]. Australian teachers have also emphasised the importance of disseminating F&N information to parents to impact food brought from home or lunch orders at school [29].

Some teachers and parents emphasised the importance of teachers’ role modelling and authority they exercise over children. These views support the evidence from Laguna et al. [50], who recently showed that American primary school students whose teachers drank water instead of sugar-sweetened beverages (SSB) in front of their classes were significantly more likely to drink water at school. However, some Australian teachers previously reported they could not model healthy eating practices very well due to their own unhealthy eating habits [29]. Similar findings have been reported in the United States (US) [51, 52]*.* In addition, the lack of FNE related knowledge and limited available teaching time were reported to be barriers to teachers becoming role models for nutrition education programs [53]. Therefore, teachers need to be equipped with teaching resources and adequate training to become good role models at school in terms of healthy eating.

Parents and teachers expressed positive views about some aspects of school food environments, such as canteens that provide healthy options, fruit provision during lesson breaks and well-implemented school food policies. The school food supply and policy can potentially reinforce nutrition education components of the school curriculum [54]. For example, the accessibility of fruit and vegetables is one of the main factors in their increased consumption among children [55]. Consistent with the literature, several teachers stated that the provision of free fruit at fruit breaks through the collaboration of some local organisations was a particular strength. This practice enables all students to access fresh fruits and vegetables at schools. These results corroborate findings by Nathan et al. [56], who discovered that schools in rural or less socioeconomically advantaged areas in Australia have the highest fruit break programme adoption rates. Parents in our study also frequently mentioned rules regarding food breaks, including nude food policy. They appreciated schools’ efforts to reinforce unpackaged and unprocessed food in lunch breaks. These reports are similar to concerns across the world on the effects of food production on climate change [57, 58] and growing interest in improving the sustainability of food consumed at school [59]*.*

### Perceived barriers to the promotion of healthy eating in primary schools

The teachers’ and parents’ reports suggest that they believe that primary schools have significant potential as an appropriate setting to promote healthy eating among children. However, they felt that schools had some constraints. Inadequate time for teaching FNE; lack of leadership, expertise and motivation of teachers; and limited resources and funding were among these limitations mentioned by both teachers and parents. Some international studies [60–62] have reported the same barriers described by American, Canadian and Sri Lankan teachers. Similarly, Australian scholars have found that teachers cited inadequate leadership and coordination, overloaded curriculum and limited availability of easily accessible FNE resources as barriers to FNE [23, 29]. Leadership has been consistently reported internationally as an important enabler of the success of the promotion of health eating in schools [63, 64]. School principals need to be involved in the promotion of healthy eating and particularly to identify a specific person or group of people to lead or coordinate efforts in the school [65]. Employment of cross-curriculum approaches was suggested as a solution in previous studies to overcome time barriers to the delivery of FNE in schools [66, 67]*.* Furthermore, it has been noted that teachers must receive appropriate content training [68] as their own education affects how much they value teaching healthy behaviours, their intentions, and self-efficacy [69]. Australian schools should be supported by external funding to maintain health-promoting activities and practices [70]. As it was argued that public schools were more disadvantaged than private schools in terms of funding they receive [71], priority should be given to public ones.

The present study also demonstrated the canteen menus and lunch order options varied widely among schools. These findings match those observed in previous studies that reported inconsistent implementation of canteen policies in Victoria and low adherence to canteen policy [72] with many discretionary food items and lack of healthy food items in the canteen menus [73]. Previous Australian studies have reported high cost of healthier food, lack of support, resources and facilities as some of the barriers to health promoting canteen policies [26, 49, 74]. Australian primary schools’ inadequate adherence to nutrition policy is not unique. Low adherence to school nutrition policy in Canada [75] and in the US [76] were also demonstrated. In addition, around 90% of countries in the European region were known to have food policies in primary schools, but the level of enforcement and the success of implementation is unknown [77].

Moreover, some parents highly criticised the abundance of unhealthy options in canteens and school events similar to parents in a previous Australian study [78] whereas some other parents regarded canteen food as a treat once a week and did not request any reform. Worsley [79] previously demonstrated these mixed views of Australian parents where only half of the parents believed that canteens should only sell healthy food. The participants’ perspectives towards school canteen food options might be affected by the operation frequency of their children’s school canteens. This is in line with Hardy et al.’s study [80], which reported that Australian primary school students who purchase lunch orders ≥2 times a week were more likely to have unhealthy weight status compared to ones purchasing ≤1 time/week. Schools need to make more effort to increase the availability of healthy options at canteens as well as at fundraising events or school fairs. Findings also suggest that canteens which operate 5 days a week, providing an abundant number of unhealthy options, should receive higher priority for any reform.

### Strengths and limitations of the current study

The study identified rich themes to depict a picture of contemporary Victorian primary schools’ role in the promotion of healthy eating through the descriptions of parents and teachers. The study did not focus on one aspect of schools’ food-related practices, but rather focused on a broad range of food issues, including school food environments, curriculum and school food policies.

Some methodological limitations must be considered when interpreting the findings of the study. First, like most qualitative studies, our study was based on convenience samples. However, it is important to note that qualitative studies do not aim to represent populations but identify themes or meanings that are likely to be present in the general population without measuring their extent. In addition, although efforts were made to recruit participants from rural areas only two parents and one teacher were actually recruited from rural areas.

Also, self-selection bias may be present; those who participated in the study may have been more interested in the study’s subject than other parents. Exploratory studies often suffer from self-selection bias, so it should be borne in mind that the views of the participants may not reflect those of others who may have no opinions on the study topic. Finally, further survey research is required to examine the prevalence of these identified themes in the general population as well as the interrelationships between the themes within various demographic groups in the general population.

## Conclusion

Parents and teachers identified the primary school as a well-positioned setting to support the development and practice of healthy dietary behaviours and food skills. They acknowledged schools’ strengths in promoting healthy eating through FNE programs, reaching the wider community, role modelling and creating healthy food environments. However, several findings suggest that schools need support and reform to overcome existing barriers such as time restrictions, financial constraints and limited educator knowledge. Some parents’ and teachers’ criticism of unhealthy school food environments highlights inconsistencies among schools and demonstrates the need for mandatory policies or school health policy implementation audits. The findings have implications for health policymakers as they develop and adopt strategies to establish health-promoting primary schools.

## Supplementary Information

**Additional file 1.**

## Data Availability

The datasets used and/or analysed during the current study are available from the corresponding author on reasonable request.

## Abbreviations

FNE: Food and Nutrition Education
SSB: Sugar-sweetened beverages
US: United States
