# Supplementary material for: Parents’ and teachers’ views of the promotion of healthy eating in Australian primary schools
Source: BMC Public Health. 2021 Oct 5;21:1788. doi: 10.1186/s12889-021-11813-6 (PMC8491384; doi:10.1186/s12889-021-11813-6)
Supplement: Supplementary file 1 — Additional file 1. [file 12889_2021_11813_MOESM1_ESM.docx]

This supplementary file is provided to give a brief explanation for the Leximancer analysis used to enhance the credibility of the findings of the Nvivo analysis. The concepts and themes automatically identified by Leximancer from parents’ and teachers’ interview transcripts can be observed on the concept maps below.

***Perceived strengths of primary schools in the promotion of healthy eating***


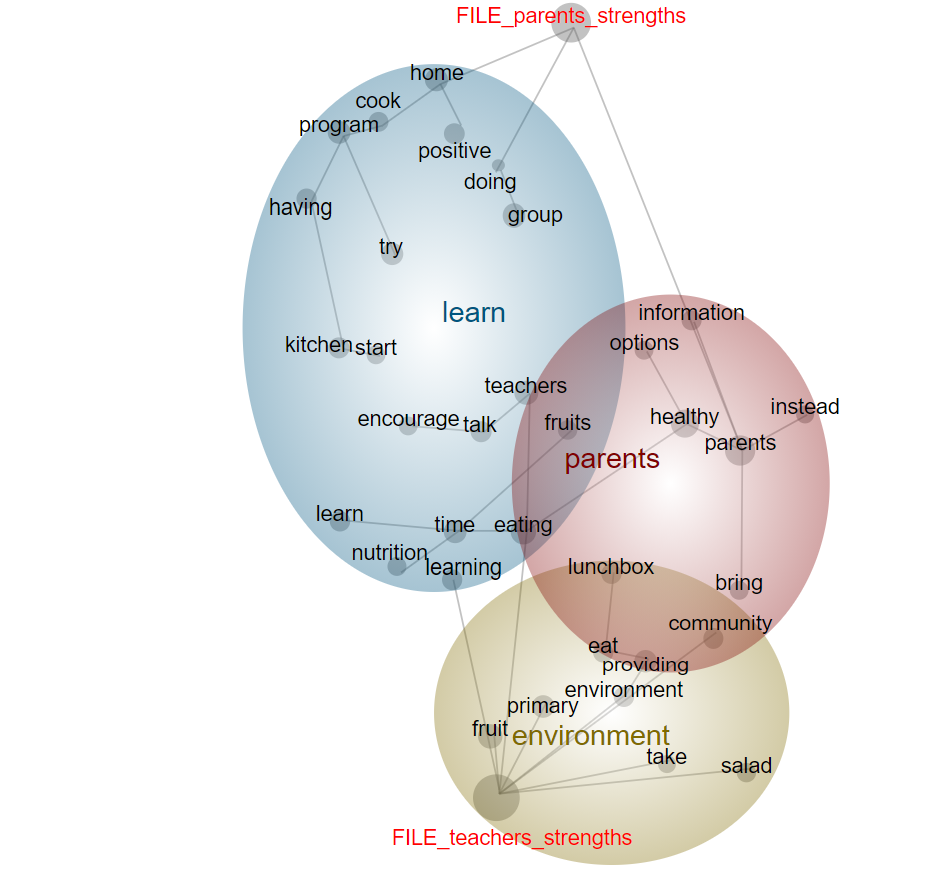


Delivery of food and nutrition education programs emerged in the ‘learn’ theme in which parents and teachers talked about the programs available at schools such as kitchen and garden programs. The ‘teachers as role models and the authority of schools’ theme was also identified under the ‘learn’ theme and was evident through the close proximity of the concepts ‘encourage’, ‘talk’ and ‘teachers’. Under the ‘parents’ theme, participants discussed being community based and reaching parents as a strength. The ‘environment’ theme covered parents’ and teachers’ comments about healthy school food environments which included fruit breaks, rules over lunch boxes and the food options at schools.

***Perceived weaknesses of primary schools in the promotion of healthy eating***


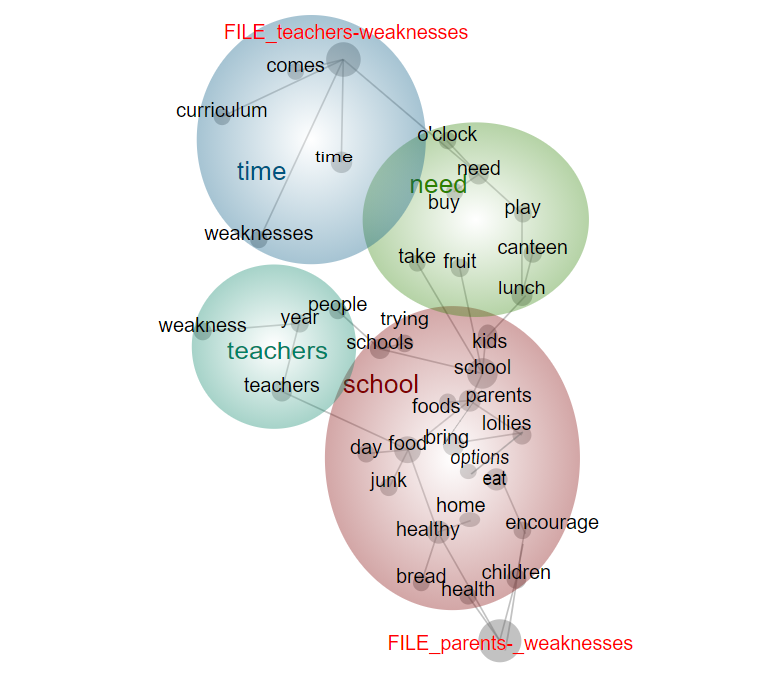


As it can be seen in the concept map, ‘time’ emerged as a theme and its close proximity to the teachers file tag indicated its more frequent mention by teachers. Under the ‘teacher’ theme, participants discussed teachers’ lack of knowledge, expertise and motivation. Unhealthy school food environment was discussed under the theme ‘school’. The concepts ‘lollies’ and ‘junk’ can also be observed under this relevant theme. Under the theme ‘need’, participants discussed the insufficient resources of schools.
